# Humic acid biosynthesis and bacterial community evolution during aerobic composting of rice straw

**DOI:** 10.1007/s00253-023-12994-3

**Published:** 2024-01-26

**Authors:** Yuwei Gao, Shuai Liu, Nan Wang, Yong-Zhong Wang

**Affiliations:** Key Laboratory of Biorheological Science and Technology (Chongqing University), College of Bioengineering, Ministry of Education, Chongqing University, Chongqing, 400030 China

**Keywords:** Humic acid, Rice straw, Composting, Microbial communities, Aerobic fermentation

## Abstract

**Abstract:**

In this study, the effects of inoculum ratio, substrate particle size and aeration rate on humic acid (HA) biosynthesis during aerobic composting of rice straw were investigated, respectively. The contents of total organic carbon, total nitrogen and HA, as well as lignocellulose degradation in the composting were evaluated, respectively. It is found that the maximal HA yield of 356.9 g kg^−1^ was obtained at an inoculum ratio of 20%, a substrate particle size of 0.83 mm and an aeration rate of 0.3 L·kg^−1^ DM min^−1^ in the process of composting. The changes of microbial communities and metabolic functions at different stages of the composting were also analyzed through high-throughput sequencing. The result demonstrates that Proteobacteria, Firmicutes, Bacteroidetes and Actinobacteria were the dominant phyla and their relative abundance significantly varied over time (*p* < 0.05), and *Rhizobium*, *Phenylobacterium*, *Pseudoxanthomonas* and *Paenibacillus* were positively related to HA content in the compost. Furthermore, the metabolic function profiles of bacterial community indicate that these functional genes in carbohydrate metabolism and amino acid metabolism were involved in lignocellulose biodegradation and HA biosynthesis. This work may be conducive to explore new regulation strategy to improve bioconversion efficiency of agricultural residues to applicable biofertilizers.

**Key points:**

*• Temperature, pH, TOC, TN and C/N caused a great influence on humic acids synthesis*

*• The succession of the microbial community during the composting were evaluated*

*• The metabolisms of carbohydrate and amino acids were involved in HA synthesis*

**Graphical abstract:**

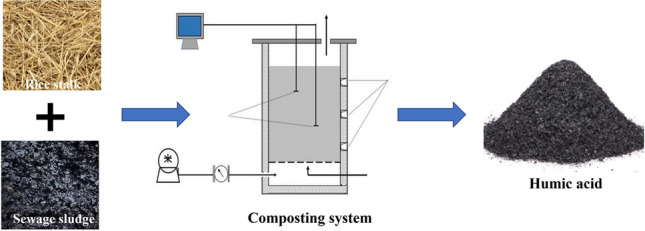

## Introduction

Currently, increasing attentions have been attracted to developing green and sustainable biofertilizers because of the concerns on serious environmental pollution resulting from large-scale application of chemical fertilizers (Cao et al. 2023). Meanwhile, about 600–900 million tons of rice straw is produced each year in the world (Zheng et al. 2014). These agricultural residues are rich in nutrients such as nitrogen, phosphorus and potassium, which can be potentially used as soil conditioner and fertilizer for crop growth (Zhang et al. 2015). However, in many countries, these agricultural residues are often burned or discarded after harvest, which causes a large sum of nutrient losses in the straws from the fields, wasting of renewable resources (Oanh et al. 2018). Therefore, it is urgent to seek alternative methods for better management of rice straw.

Composting is an environment-friendly and reliable approach for rice straw utilization, by which these cellulosic materials can be converted into humic substances in a great deal by microorganisms and used as organic fertilizer. According to temperature change during the composting, it can be divided into three phases, i.e. the mesophilic, thermophilic and mature stages (de Gannes et al. 2013). The organic fertilizers produced by composting possess high humic acid (HA) content and an abundance of microorganisms (Lv et al. 2013; Watteau and Villemin 2011). Thus, applying the biofertilizers can not only improve soil fertility to promote crop growth, but also play important roles in soil bioremediation (Zhang and He 2004) and carbon sequestration in soil (Wei et al. 2022). This has been considered a promising strategy to tackle soil erosion and improve soil nutrient content. Although the composting has been widely adopted to convert these agricultural residues into organic fertilizers, there is lack of knowledge on the link of microorganisms with HA biosynthesis (Qian et al. 2014).

In the process of aerobic composting, operating conditions such as inoculum ratio, substrate particle size and aeration rate (AR) cause significant effect on heat and mass transfer and HA biosynthesis (Li et al. 2017; Zhang et al. 2016a). An appropriate aeration can’t only carry the produced CO_2_ out of the compost, but also provide sufficient O_2_ to microbes for growth and metabolism (Bernal et al. 2009). In addition, the moisture content of cellulosic materials during composting is influenced by porosity of the packed bed and particle size of the fermentation substrate (Wang et al. 2019). Also, HA bioconversion involves many complex chemical and biological events (Zhang et al. 2016b), and is regulated by various microbial actions (Zhang et al. 2016a; Liang et al. 2020). Although fungi are the main producers of lignocellulose-degrading enzymes in the composting, the lignocellulose-degrading bacteria and nitrogen-fixing bacteria have been considered the most important microbial species of this process (Harindintwali et al. 2020). These bacteria such as *Bacillus sp* usually grow faster than fungi, and the produced cellulases are more tolerant to high temperature, heavy metal and low pH (Latt et al. 2018; Harindintwali et al. 2020; Abdel-Rahman et al. 2016). Nitrogen-fixing bacteria can catalyze the conversion of N_2_ to NH_3_ by producing nitrogenase to improve nitrogen content in compost (Jabir et al. 2018). Several nitrogen-fixing bacteria such as *Stenotrophomonas*, *Rhizobium* and *Azomonas* have been isolated in the process of decomposing straw (Latt et al. 2018; Zainudin et al. 2013). Therefore, the microbial diversities during composting bring great influence on bioconversion of lignocellulosic substrate into the biofertilizers (Jurado et al. 2014). However, there have been little investigation on the relationship of microbial community and humic acids biosynthesis.

In this work, the effects of operating conditions including inoculum ratio, substrate particle size and AR on the characteristics of HA biosynthesis were individually investigated using rice straw as composting substrate, and the microbial diversity in the compost under the optimal composting conditions was detected by by high-throughput sequencing. The dynamic behaviors of the microbial communities was then revealed, also, the bacterial metabolic functions at different composting stages were predicted by PICRUSt (Phylogenetic Investigation of Communities by Reconstruction of Unobserved States) algorithm. The relationships among the HA biosynthesis and the microbial communities, as well as the bacterial metabolic functions were discussed. This work may be conducive to explore new regulation strategy to improve bioconversion efficiency of agricultural residues to applicable biofertilizers.

## Materials and methods

### Experimental set-up and design

**Fig. 1:**
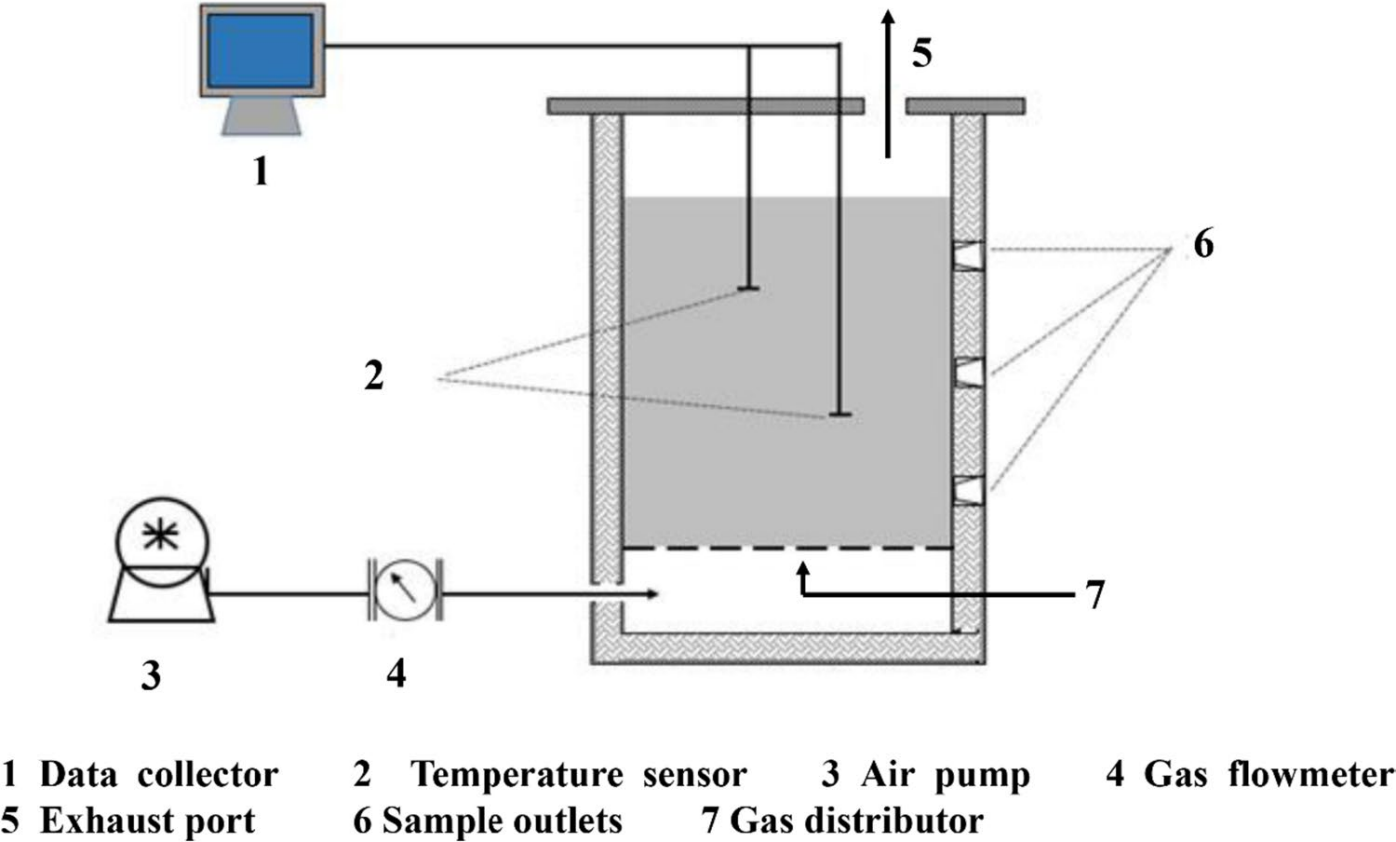
The schematic diagram of the composting system

The composting was conducted in an aerobic fermentation system, which includes a cylindrical bioreactor, an air pump and a data acquisition unit. The cylindrical bioreactor (Ø100 mm × H180 mm) was made of polymethyl methacrylate with a total volume of 1.41 L. A gas distributor with 5 mm apertures was installed at the bottom of the bioreactor. The air was pumped into the bioreactor by an air pump (ACO-003, Zhoushan, China), and a data acquisition unit (Agilent 34,972 A, Beijing, China) was used to measure the compost temperature (environmental temperature was controlled at about 32 °C). A schematic diagram of the testing system is shown in Fig. 1.

### Materials and composting

The used rice straw was taken from a local farmland of Chongqing City in China. The sewage sludge with the mixed liquor suspended solids of 40% and C/N ratio of 9.17 was collected from a local paper mill’s wastewater treatment plant. The specific physicochemical characteristics of the raw materials are stated in Table 1. The effects of inoculum ratio, substrate particle size and AR on the contents of total organic carbon (TOC) and total nitrogen (TN), lignocellulose contents and HA yield in compost were evaluated, respectively. The substrate moisture was maintained at about 60% by supplementing distilled water during the composting in all treatments, and the fermentation substrate was turned over every 5 days. Each test was conducted in triplicates. The samples for HA content detection and DNA extraction were collected on 0, 5th, 10th, 16th, 22nd, 29th and 36th day of composting, respectively.

**Table 1:** The basic physical and chemical properties of the composting materials

Materials	Moisture (%)	TOC (g kg^−1^)	TN (g kg^−1^)	C/N	Volatile solid (%)
Sewage sludge	82.68 ± 1.56	189.97 ± 1.96	20.27 ± 1.53	9.17	50.61 ± 1.47
Rice stalk	6.64 ± 0.11	354.22 ± 3.77	4.40 ± 0.15	80.44	80.96 ± 3.63

^a^ Values are mean±standard deviation (*n* = 3)

### Analysis methods

The data acquisition system was used to regularly detect the temperatures of the compost and the surrounding environment.

The fermented substrate was dried at 100 ± 5 °C in an oven (DHG-9055 A, Shanghai, China) till constant weight and the moisture content of the substrate was calculated. TOC was determined according to Chinese National Standard (NY525-2012). The wet combustion method (potassium dichromate oxidation method) was adopted for the calculation of TOC content. The calculation formula is shown as

1 $$\omega = \frac{{\mathrm{c(V}}_0-\mathrm{V}) \times 0.003 \times 100 \times 1.5 \times 1.724 \times \mathrm{D}}{{\mathrm{m(1-X}}_0\mathrm{)}}\%$$

where ω is the content of TOC (%), c is the molar concentration of the ferrous sulfate standard solution (mol/L). V_0_ and V represent the volumes of the standard solution consumed in blank test (mL) and in sample determination (mL), respectively. 0.003, 1.5 and 1.724 show the molar mass of one-fourth carbon atom (g/mol), a oxidation correction coefficient and a conversion coefficient of organic carbon to organic substrate, respectively. M and X_0_ refer to the mass of the air-dried sample (g) and the moisture content of the air-dried sample (g), individually. D is a ratio of constant volume versus partition volume (Here is 250/50).

TN in the sample was detected according to Chinese National Standard (GB T 8572 − 2010). The titrimetric method after distillationwas adopted for the determination of TN content. The C/N ratio was calculated based on the values of TOC and TN. The lignocellulose content was detected according to the method of Vansoest et al. (1991). HA in the fermented substrate was extracted and detected according to the literature (Zhou et al. 2014) and HA content was calculated according to the fellow formula.

2 $$\mathrm{HA} \ (\%)=\frac{{0.003(\mathrm{V}_0}-\mathrm{V)M}}{\mathrm{C} \times \mathrm{G}} \times \frac{\mathrm{a}}{\mathrm{b}} \times {100\%}$$

Where V_0_ and V are the volumes of ferrous ammonium sulfate solution consumed in blank and in the sample, respectively. M is the concentration of standard solution of ferrous ammonium sulfate; a and b are the total volume of the sample solution and the volume of the sample solution used for measurement, respectively. G and C refer to the sample weight and the carbon coefficient of HA in the samples (Here, C is 0.58), respectively.

### DNA extraction and Illumina MiSeq sequencing

The total DNA of the sample was extracted using E.Z.N.ATM Mag-Bind Soil DNA Kit according to manufacturer’s instructions. The purity of extracted genomic DNA was detected using 1% (w/v) agarose gel electrophoresis. The V3-V4 region of l6S rRNA genes were amplified using two primers 515 F (5′-GTGCCAGCMGCCGCGGTAAT-3′) and 806R (5′-GGACTACHVGGGTWTCTAA-3′). PCR reactions were carried out in 30 µL of the mixture with 2 × Taq master Mix (15 µL), 10 µM primer 515 F (1 µL), 10 µM primer 806R (1 µL) and 10 ng templates DNA. In the process of PCR, DNA was denatured at 94 °C for 5 min, followed by 30 cycles at 94 °C for 40 s, 58 °C for 40 s, 72 °C for 60 s and finally 1 cycle at 72 °C for 5 min. The PCR products were verified using agarose gel electrophoresis (2%, w/v) and purified using Agencourt AMPure XP system (Beckman Coulter Inc, Brea, CA, USA), then, were entrusted to Biomarker Technologies Corporation (Beijing) for high-throughput sequencing to reveal the bacterial community structure. QIIME software (Version 1.8.0) was used to cluster Tags at a similar level of 97% to obtain OTUs. The OTUs were annotated based on the Silva (bacteria) taxonomy database (Edgar 2010). The microbial community indicators, including ACE, Chao1, Simpson, Shannon and Good’s Coverage indices, were calculated using Mothur software (version v.1.30), and the metabolic function profiles of the microbial community were predicted using PICRUSt algorithm. The raw reads have been uploaded to the NCBI Sequence Read Archive (SRA) database (accession number: PRJNA971886).

### Data analysis

Each parameter test was carried out by three parallels and the experimental results were expressed as the mean ± standard deviation. A level of *p* < 0.05 in variance analysis was statistically considered significance. The correlation network analysis was performed using the sparcc algorithm (Friedman and Alm 2012), and spearman correlations among physicochemical parameters, HA content and bacterial diversity were obtained by GraphPadPrism (version 8.0.2). Heatmap analysis was conducted by R software (Version 2.15.3).

## Results

### Effect of the inoculum ratio on decomposition of rice straw and humic acid formation

In this experiment, the inoculum ratio was set at 5%, 10%, 15%, 20% and 25% (v/v). The initial substrate particle size and AR were fixed at 0.38 mm and 0.5 L·kg^−1^ DM min^−1^, respectively. The substrate moisture was maintained at about 60% by adding distilled water during the composting, and the environmental temperature was controlled at about 32 °C.

**Fig. 2:**
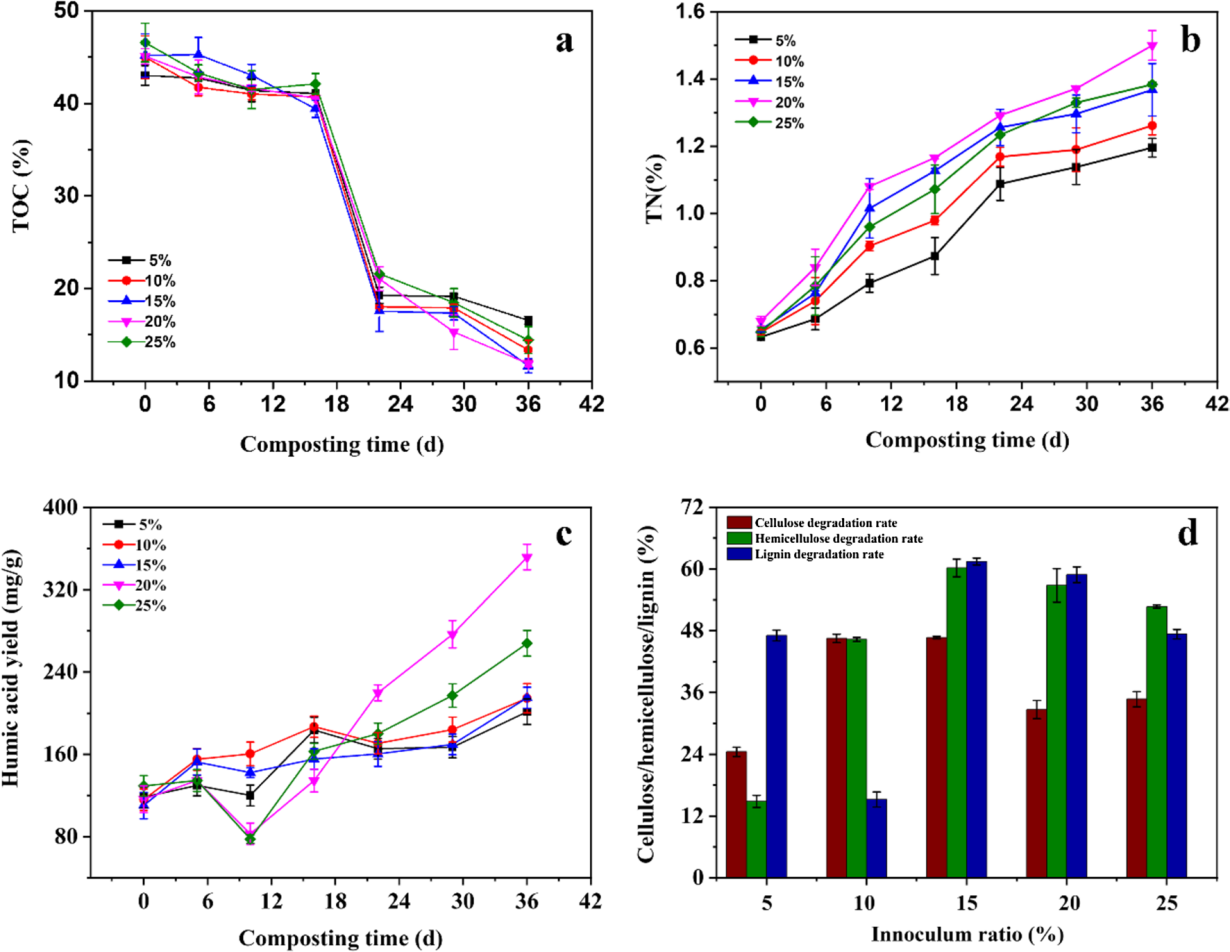
Effect of inoculum ratio on TOC (**a**), TN (**b**), HA yield (**c**) and lignocellulose degradation rate (**d**) during the composting. The abbreviations of total organic carbon, total nitrogen and humic acid are TOC, TN, HA, respectively. The same below

Figure 2 depicted the TOC, TN, HA content and lignocellulose degradation rate at different inoculum ratios, respectively. As shown in Fig. 2a, it always decreased during the composting in all treatments. Li et al. (2019) reported that in the process of composting, the organic carbon in fermentation substrate could be adopted as carbon source for microbial proliferation and aerobic fermentation. TOC content is a clear indicator of the mineralization of easy degradation fractions in organic matter (Awasthi et al. 2015). Here, the TOC content slightly decreased at the initial stage of the composting (0-16th days) in all inoculum ratios, then, decreased sharply on 16th -22nd days. This means that the metabolic activities of thermophilic microorganisms were weak at the initial composting, then significantly increased after acclimating to the fermentation environment, which was consistent with the result of Huang et al. (2017). After that, the TOC content almost became relatively constant in all treatments, it shows that the growth and metabolism of these microorganisms became in equilibrium state. Furthermore, at the end of the composting, the TOC contents at 15% and 20% inoculum ratios were 11.64% and 11.86%, respectively, which were lower than the two groups with inoculum ratios of 5% and 10%.

The TN content increased steadily during the composting in all treatments (Fig. 2b). This result means that the composting could effectively promote the degradation of nitrogen-containing organic substrate such as rice straw (Zhang et al. 2016c). At the end of the composting, the highest TN content of 1.50% was obtained at 20% inoculum ratio, while the TN content was only 1.19% at 5% inoculum ratio. This indicates that the decomposition of nitrogenous compounds was limited at 5% inoculum ratio due to a low biomass. C/N ratio is an important parameter of evaluating maturity and stability of the compost. Studies have shown that fermentation substrate fully matured when C/N ratio was below 20 during composting (Guo et al. 2012). At the end of the composting, the C/N ratio decreased to 13.74, 10.59, 8.47, 7.91 and 10.53 at 5–25% inoculum ratios, respectively. The minimal C/N ratio of 7.91 was obtained at 20% inoculum ratio, which implies that the highest degree of maturity was achieved in this group.

The contents of HA increased over time in all treatments (Fig. 2c). It was quite likely that the decomposed organic compounds were converted into HA by these microbes (Jouraiphy et al. 2005). At the end of the composting, the HA content initially ascended with an increase in the inoculum ratio to 20% and then significantly descended to 276.93 mg g^−1^ with further increase in inoculum ratio to 25%. A peak value of HA content, 351.72 mg g^−1^, was achieved at 20% inoculum ratio, which indicates that the treatment with 20% inoculum ratio could reach the high level of humification. This is consistent with the result from Awasthi et al. (2015).

In addition, the degradation rates of lignin, cellulose and hemicellulose in the testing groups at 15% and 20% inoculum ratios were significantly higher than those of other groups (Fig. 2d). This can be explained by that the suitable inoculum ratio could cause rapid decomposition of organic compounds to form HA (Zhou et al. 2014).

### Effect of substrate particle size on decomposition of rice straw and humic acid formation

Here, the effect of substrate particle size (4.75 mm, 2.36 mm, 0.83 mm, 0.38 mm and 0.25 mm) on HA fermentation was investigated at an aeration rate of 0.5 L·kg^−1^ DM min^−1^, an inoculum ratio of 20% and room temperature.

**Fig. 3:**
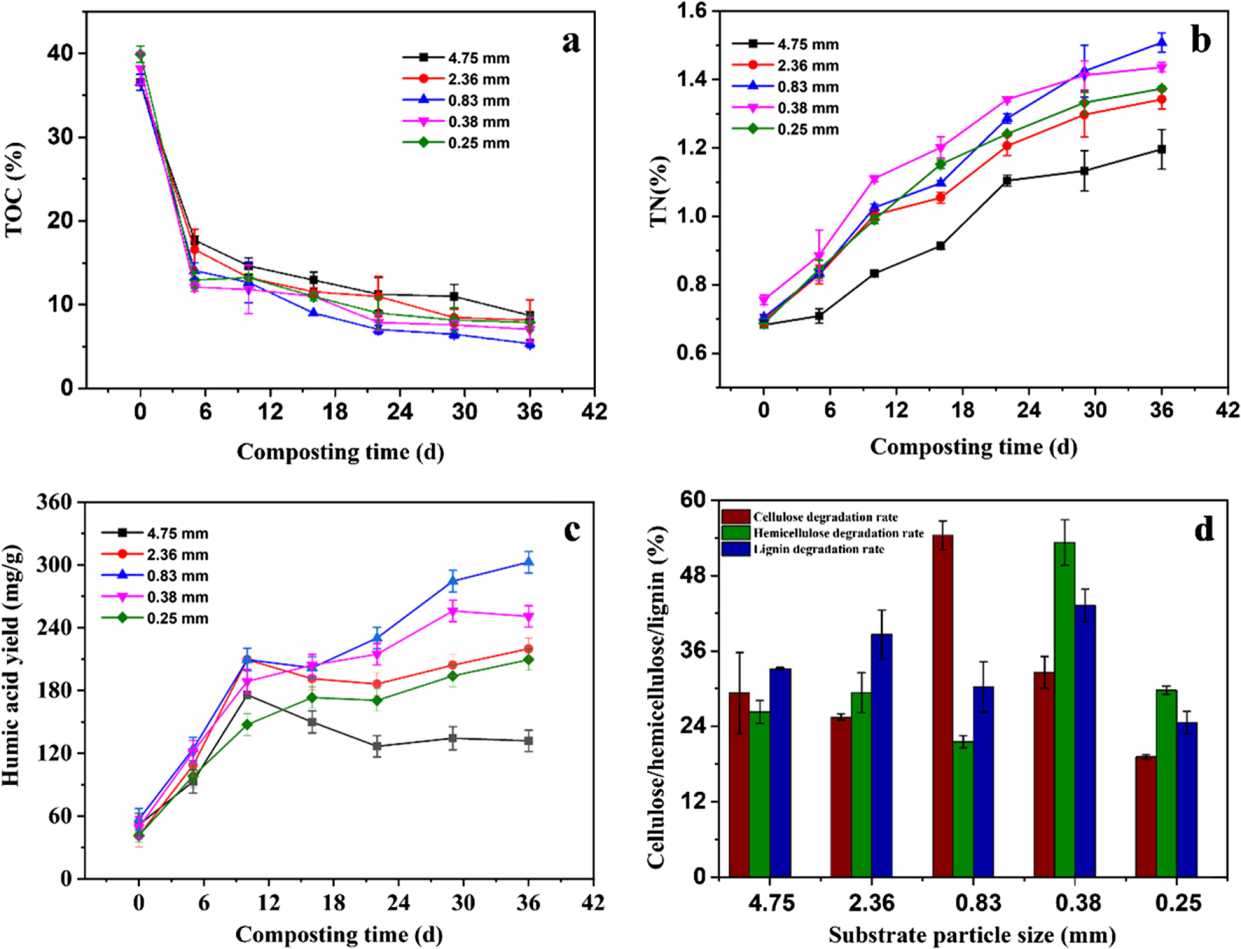
Effect of substrate particle size on TOC (**a**), TN (**b**), HA yield (**c**) and lignocellulose degradation rate (**d**) during the composting

As illustrated in Fig. 3a, TOC content rapidly descended on 0-5th days of the composting in all runs, following that, the TOC content slowly decreased. It may be induced that the easily-degradable organic matter at the mesophilic stage of the composting was first utilized by microorganisms, then, complex-structure organic compounds such as macromolecular substrate were decomposed. As a result, the TOC content went down rapidly at the initial stage of cultivation, then descended slowly at the thermophilic and mature stages. At the end of the composting, the minimal TOC content of 5.34% was obtained at the substrate particle size of 0.83 mm, while the maximal TOC content of 8.72% was observed at the substrate particle size of 4.75 mm (Fig. 3a). It demonstrates that more organic matter was decomposed by these microbes in the compost of the rice straw with the substrate particle size of 0.83 mm. This can be considered that at the substrate particle size of 0.83 mm, the specific surface area of straw was in favor of mass transfer of O_2_ in the compost and microbial cell adsorption on the substrate surface.

The TN content increased steadily during the composting in all testing groups (Fig. 3b). At the end of the composting, the TN content showed a gradual rise with a decreasing substrate particle size from 4.75 mm to 0.83 mm, and then a slight decrease with further decrease in substrate particle size to 0.25 mm. The maximal TN content (1.51%) was achieved at the substrate particle size of 0.83 mm, suggesting that the substrate particle size was suitable for the stable degradation of N-containing compounds during the composting. The result is consistent with that of Zhang et al. (2015). However, the TN content slightly decreased to 1.37% at the substrate particle size of 0.25 mm. The maximal maturity could be achieved using the substrate with 0.83 mm substrate particle size.

The HA yields gradually increased over time in all test groups during the composting. At the end of the composting, the HA yield demonstrated an increasing trend with an initial decrease in the substrate particle size from 4.75 mm to 0.83 mm, and a subsequent decrease trend with further decrease in the substrate particle size to 0.25 mm. The maximal yield of HA of 302.59 mg g^−1^ was achieved at the substrate particle size of 0.83 mm, the minimal HA yield of 131.90 mg g^−1^ was observed at the substrate particle size of 4.75 mm (Fig. 3c). The result shows that HA biosynthesis in the 5 test groups was visibly affected by the substrate particle size of the compost.

By the end of the fermentation, the maximal degradation rate of cellulose (54.43%) was obtained at the substrate particle size of 0.83 mm (Fig. 3d), indicating that most of the cellulose could be hydrolyzed by microbes. However, the maximal degradation rates of hemicellulose and lignin (53.28% and 43.25%) were observed in the test group with substrate particle size of 0.38 mm, respectively. It can be considered that the difference in microbial diversity affected the performances of microorganisms to degrade the lignin, hemicellulose, and cellulose components in rice straw, causing differences in degradation rates of different lignocellulosic components.

The abovementioned result demonstrates that a suitable substrate particle size was advantageous to mass transfer of O_2_, cell adsorption and subsequent degradation of substrate, resulting in change of microbial community structure during the composting. In this work, the optimal substrate particle size for HA biosynthesis was 0.83 mm.

### Effect of aeration rate on decomposition of rice straw and humic acid formation

Here, based on the previous experimental results, the effect of the aeration rate on HA biosynthesis was tested, which was set at 0.1, 0.3, 0.5, 0.7 and 0.9 L·kg^−1^ DM min^−1^ (denoted as AR0.1, AR0.3, AR0.5, AR0.7 and AR0.9). The initial substrate particle size and inoculum ratio were set at 0.38 mm and 20%, respectively.

**Fig. 4:**
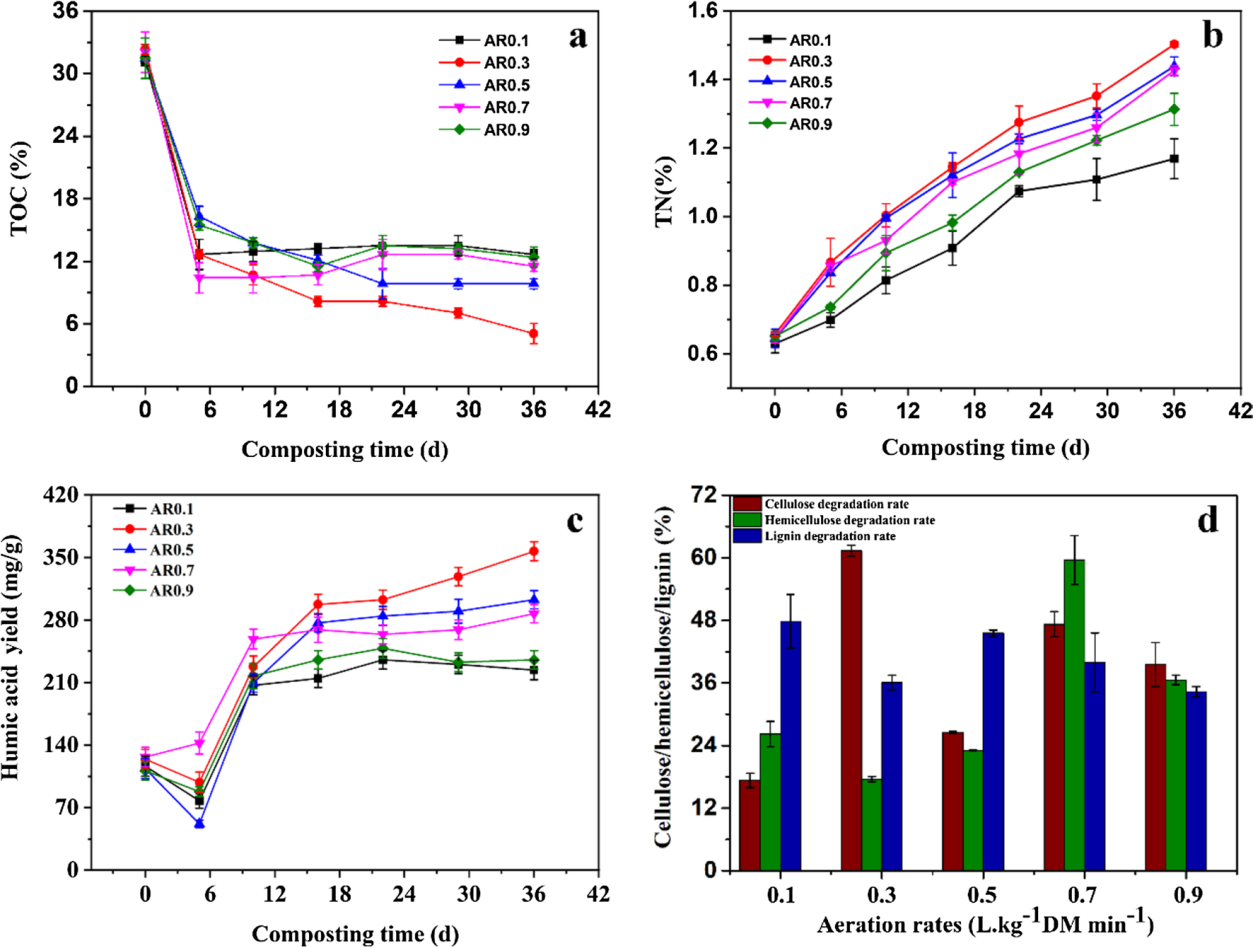
The changes in TOC (**a**), TN (**b**), HA yield (**c**) and lignocellulose degradation rate (**d**) during the composting at different aeration rates. The abbreviations are total organic carbon (TOC), total nitrogen (TN) and humic acid (HA)

As shown in Fig. 4a, the contents of TOC gradually decreased with the prolonging fermentation time in all test groups. At the end of the composting, the TOC content initially decreased with a rise in AR to 0.3 L·kg^−1^ DM min^−1^, then increased with further rise in AR to 0.9 L·kg^−1^ DM min^−1^. The loss of TOC in AR 0.3 was the maximal than other treatments, indicating that the proper mass transfer rate of oxygen resulted in the highest decomposition degree of organic matter. Hernandez et al. (2006) obtained a similar result. However, excessive AR (AR0.5-0.9) could cause a decrease in culture temperature of the compost and evaporation of more water, resulting in the low microbial activity and low humification degree. The result is consistent with previous report on co-composting of sewage sludge and corn stalk (Li et al. 2017; Harindintwali et al. 2020).

On the contrary, TN contents increased in all treatments (Fig. 4b). This phenomenon may be ascribed to the decomposition of protein and other N-containing compounds in the humification process. At the end of the composting, the maximal TN content was obtained at AR0.3 (1.50%). The TN contents in the AR 0.5–0.9 groups were 1.44%, 1.43% and 1.31% at the same composting time. Moreover, the minimal TN content (1.17%) was observed at AR0.1, meaning that a low mass transfer rate of oxygen affected the microbial activity. Furthermore, the minimal C/N (3.40) among all the treatments was observed in AR0.3, indicating that the highest maturity degree was reached in the treatment of AR0.3 group.

As depicted in Fig. 4c, HA content slightly decreased at 0-5th days in all treatments. This is quite likely that the HA molecule in the fermentation substrate was partially utilized by microbial cells. Subsequently, the HA content increased significantly in all treatments and reached a peak of 356.9 mg g^−1^ at AR0.3. The result may be explained by that the organic compounds were effectively decomposed and HA was efficiently synthesized (Li et al. 2017). The result suggests that the HA formation was visibly influenced by AR. In addition, the maximal degradation rate (61.38%) of cellulose was observed in treatment AR0.3 at the end of the composting (Fig. 4d). However, the maximal degradation rates of hemicellulose and lignin (59.61% and 47.85%) were observed at AR0.7 and AR0.1, respectively. It can be considered that the optimal operating parameters for HA biosynthesis included inoculum ratio 20%, substrate particle size 0.83 mm and AR0.3.

### Evolution of bacterial community during rice straw composting

#### Bacterial alpha diversity

To clearly clarify the changes of microbial community structure involved in the process of composting, the 16 S rDNA sequences in these samples from 0 day (M01), 10th day (M02), 22nd day (M03) and 36th day (M04) under the optimal operating parameters of HA biosynthesis were sequenced using Illumina MiSeq Sequencing. After quality filtering and non-specific amplification sequences, 202,064 high quality sequences and 2,229 OTUs above 97% similarity were obtained in these composing samples (Table 2). Both of the Chao1 index and the Shannon index reveals that the highest microbial abundance was reached in M04 among the four samples, while the lowest level of microbial diversity was observed in M01. This may be ascribed to that these microbes which could efficiently decompose lignocellulosic substrate rapidly proliferated at the mature stage of the composting.

**Table 2:** Richness and diversity index

Sample	Sequences	OTU	Shannon	ACE	Chao1	Coverage	Simpson
M01	45,453	429	4.06	460.53	469.53	0.9985	0.04
M02	56,921	491	4.32	538.44	543.87	0.9985	0.03
M03	47,038	635	4.93	655.89	667.69	0.9986	0.02
M04	52,652	674	5.35	692.89	695.03	0.999	0.01

#### The dominant phyla during the composting

**Fig. 5:**
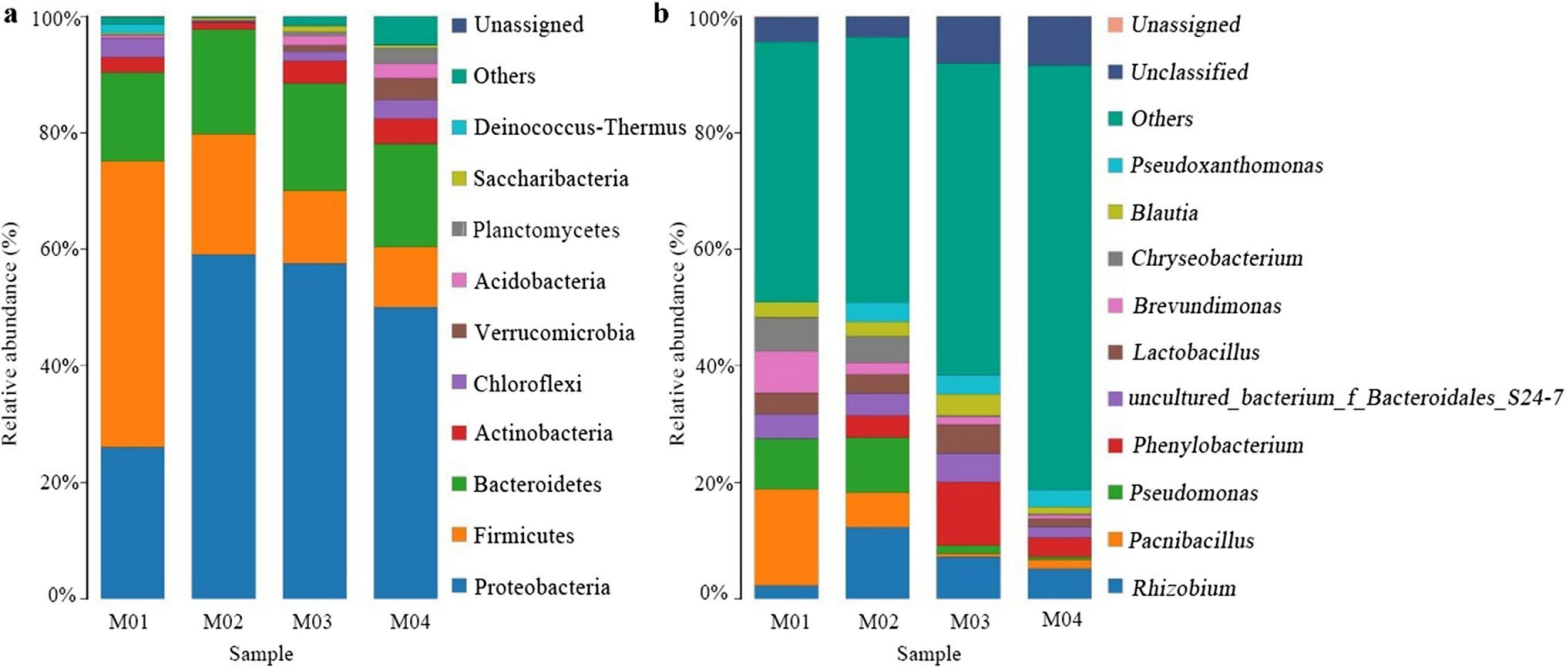
Phylum-level (**a**) and genus-level (**b**) of microbial communities during composting of rice straw

The functional microorganisms involved in the HA biosynthesis was further analyzed through the relative abundance of the bacterial communities at the phylum level. The top 10 species in the abundance level were displayed in Fig. 5a, “Unclassified” represented the species that have not been taxonomically annotated and the species after the top 10 species in the abundance level were merged into “Others”. Proteobacteria, Firmicutes, Bacteroidetes and Actinobacteria were considered the dominant phyla in the composting, and their relative abundances were 26.1–59.1%, 10.4–49.1%, 15.3–18.4% and 1.2–4.3%, respectively (Fig. 5a). Also, these bacteria at phylum level were observed in other waste composting (Wang et al. 2017). The relative abundances of Proteobacteria, Bacteroidetes and Actinobacteria showed an increasing trend with the progress of the composting. Proteobacteria was a predominant taxon during the composting which didn’t only degrade lignocellulose, but also mineralize nitrogen-containing organics (Qiu et al. 2019). Bacteroidetes was also a dominant phylum, which was considered to be involved in biogeochemical carbon cycling and heavily proliferate at the thermophilic stage of composting (Mao et al. 2018), resulting in degradation of organic carbon. Firmicutes demonstrated the highest abundance level in M01 in this work. Ali et al. (2019) found that Firmicutes could decompose lignocellulosic crop residues through the secreted lignocellulolytic enzymes. However, in this work, the relative abundance of Firmicutes sharply decreased from 49.1 to 10.4% over time of the composting. It suggests that with the progress of fermentation, most of the organic substrate might be utilized to synthesize HA.

#### The dominant genera during the composting

At the genus level, a total of 744 bacterial genera were found in our samples, and the changes of relative abundance at the bacterial genera level almost were in accordance with those at the phylum level (Fig. 5b). With the progress of composting, the relative abundance of *Rhizobium* (phylum Proteobacteria) increased, reaching the maximal value of 7.2% on the 10th day (M02). Here, the relative abundance of *Paenibacillus* (phylum Firmicutes) gradually decreased from 16.6% on the first day (M01) to 0.59% on 22nd day (M03), the declination of this genus can be ascribed to a drop in lignocellulose content. Additionally, *Pseudomonas* occurred in M02 group, and its abundance level was maintained relatively constant till the end of the composting. *Phenylobacterium* occurred in M02 group, and the maximal abundance was observed in M03 group. *Pseudoxanthomonas* was observed in M02 group and its abundance level was always maintained relatively stable. In this work, the relative abundance of *Pseudomonas* (phylum Proteobacteria) sharply decreased from the 10th day (M02) to the 22nd day (M03), which was ubiquitous in lignocellulosic composting systems, and also involved in straw degradation (Wei et al. 2018).

### The relationships of microbial community with physico-chemical characteristics and humic acid biosynthesis

**Fig. 6:**
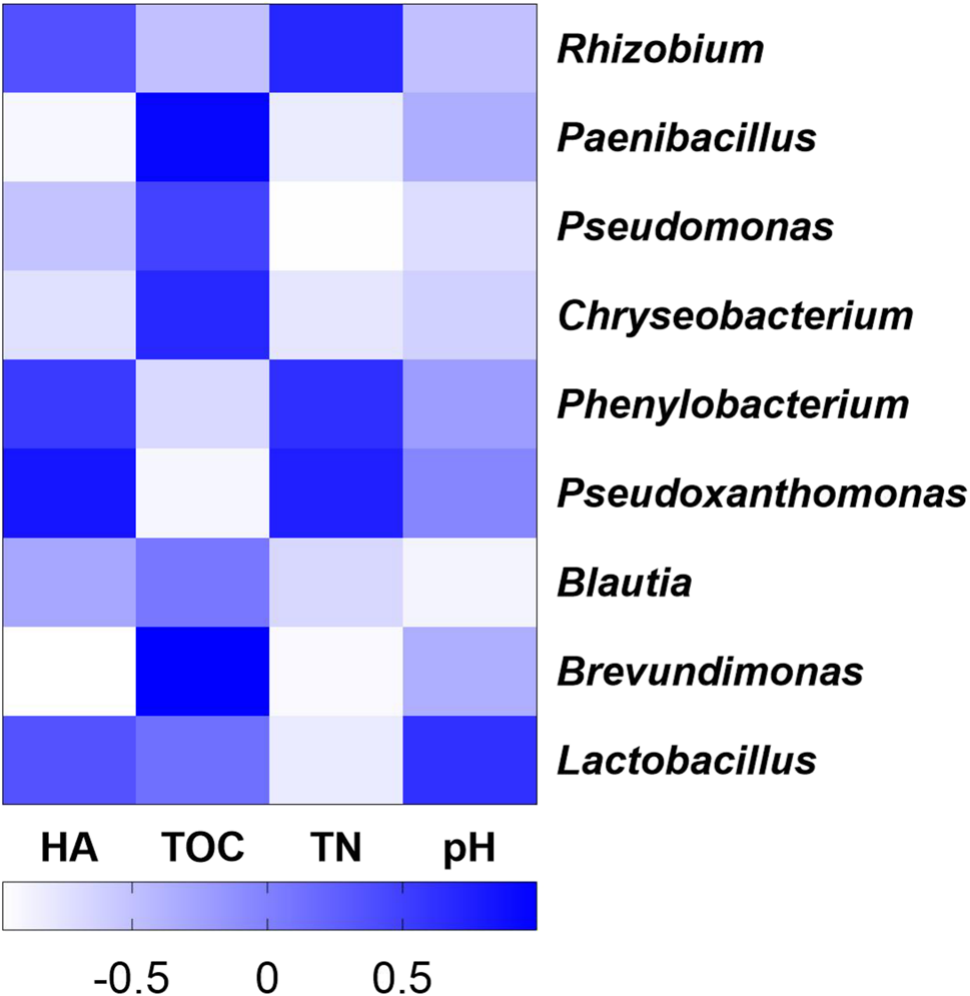
Spearman correlation analysis. The spearman correlation coefficient is r, positive correlation (r > 0), negative correlation (r < 0)

Spearman correlation analysis was used to evaluate the relationships among the dominant bacterial genera and HA content, as well as physicochemical charateristics. The top 9 bacterial genera with the highest relative abundance were considered as the dominant bacterial genera, respectively. It is found that *Rhizobium*, *Phenylobacterium*, *Pseudoxanthomonas* and *Paenibacillus* were positively related to HA content, whereas *Pseudomonas*, *Chryseobacterium*, *Blautia*, *Brevundimonas* and *Lactobacillus* were negatively related to that at the genera level (Fig. 6). Among these microorganisms, *Rhizobium*, *Phenylobacterium, Pseudoxanthomonas* and *Paenibacillus* possibly participated in the biosynthesis of HA. Notably, *Brevundimonas* showed highly significant positive correlation with TOC content (*p* < 0.01), but an opposite phenomenon was observed between it and HA content. This indicates that *Brevundimonas* mainly participated in decomposing organic matter rather than in HA biosynthesis. Correlation analysis also indicated that *Rhizobium*, *Phenylobacterium* and *Pseudoxanthomonas* were positively related to TN, whereas other dominant bacterial genera were negatively related to that. Many researchers stated that *Phenylobacterium* possessed an important effect on degradation of proteinaceous substances, resulting in a high nitrogen content in HA (Li et al. 2019; Qiu et al. 2019). However, only *Lactobacillus* was positively related to pH. Li et al. (2020) found that *Lactobacillus* could improve the quality of the final product of sheep manure composting through producing organic acids, special enzymes, bacteriocin and other substances (Biasato et al. 2020). The above result suggests that the dominant bacterial genera could cause effective influence on the humification in the process of the composting in this work.

### Bacterial metabolic function during rice straw composting

**Fig. 7:**
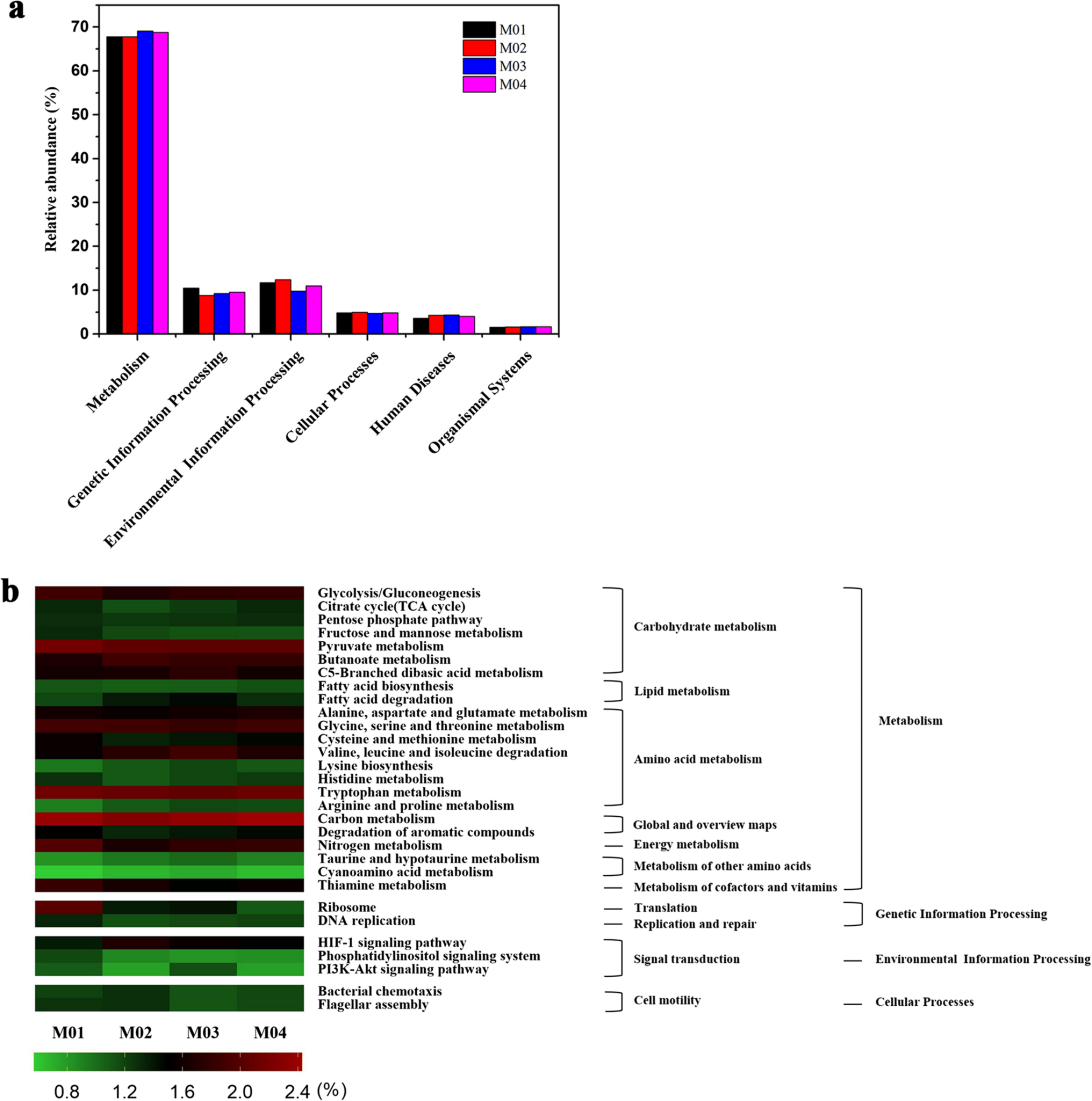
Metabolic function profiles during composting process of rice stalk in aerobic. (**a**) Biochemical metabolic pathways, (**b**) KEGG ortholog function categories of the 30 most metabolic functions on Level 3

Among these predicted protein sequences annotated with KEGG pathway in the five samples, 67.80–69.09% of them belonged to the metabolism group, 8.83–10.46% to genetic information processing, 9.84–12.42% to environmental information processing, 4.74–4.98% to cellular processes, 3.59–4.38% to human diseases and 1.58–1.70% to organismal systems (Fig. 7a). According to KEGG ortholog function categories of the major metabolic functions on Level 2, the metabolisms of carbohydrate, lipid, amino acid, other amino acids, energy, cofactors and vitamins, as well global and overview maps, were the main pathways in the cluster of metabolisms. In addition, translation, replication and repair were the major pathways in the cluster of genetic information processing. Signal transduction was the dominating pathway in the cluster of environmental information processing. Cell motility was the main pathway in the cluster of cellular processes, this could promote degradation of more organic matter and HA biosynthesis.

Furthermore, the KEGG ortholog function categories of the major metabolic functions on Level 3 were analyzed in detail (Fig. 7b). For carbohydrate metabolism, glycolysis/gluconeogenesis, fructose and mannose metabolism and pyruvate metabolism showed higher relative abundances levels in M0 than those of other samples. Additionally, as for amino acid metabolism, the abundance of these genes involved in glycine, serine and threonine metabolisms, cysteine and methionine metabolisms, and tryptophan metabolism decreased at the stages of M01-M03, while slightly increased at the fourth stage (M04). On the contrary, the abundance of these genes related to valine, leucine and isoleucine degradation, lysine biosynthesis, arginine and proline metabolism increased during the composting. The protein sequences related to metabolism of cofactors and vitamins at different stages of the composting accounted for 1.49–1.82% of the total protein sequences, this might reduce N loss in the compost.

## Discussion

As mentioned above, operation conditions caused visibly influence on the humification. An appropriate inoculum ratio can promote the effective degradation of organic matter and the rapid heating of compost. As shown in Fig. 2, at the end of the composting, the TOC contents at 15% and 20% inoculum ratios were lower than the two groups with inoculum ratios of 5% and 10%. This means that the high microbial activities were reached at the inoculum ratio of above 15% and more organic substrate was decomposed. At the inoculum ratio of 20%, a peak value of HA content was achieved, which means that the metabolic activities of microorganisms were enhanced, resulting in more lignocellulose being converted to HA and reaching the high level of humification.

The structural integrity of lignocellulose and mass transfer efficiency in compost are also influenced by substrate particle size (Zhang et al. 2016a). In this work, the TN content and HA yield showed an initial increase and a subsequent decrease with a decreasing substrate particle size from 4.75 mm to 0.25 mm (Fig. 3). This phenomenon means that at a small substrate particle size more cells could be adsorbed on the substrate surface due to a large specific surface area of these substrate particles, then, more organic substrate was decomposed. Furthermore, the substrate particle size caused a change in porosity of the compost, low compost porosity resulting from small substrate particle size easily caused a reduced mass transfer rate of oxygen, which was not conducive to microbial degradation of nitrogenous compounds (Guo et al. 2019). Likewise, a high compost porosity resulting from a high substrate particle size went against the cell adsorption and decomposition of rice straw (Hernandez et al. 2006). At the end of the composting, the maximal degradation rate of cellulose was obtained at the substrate particle size of 0.83 mm.

Furthermore, AR can bring significant influence on HA formation during composting (Bernal et al. 2009). The TOC content initially decreased with a rise in AR, then increased with further rise in AR (Fig. 4). However, TN content showed an increasing trend. It can be considered that the excessive AR could cause loss of more water in compost, resulting in a slow degradation of nitrogen-containing organic compounds by microorganisms. With an initial increase in aeration rate, the oxygen transfer rate gradually increased, which is conducive to the growth and metabolism of aerobic microbes. While with further increase in AR, the HA content became relatively constant under the experimental conditions. AR mainly affects the activities of composting microorganisms through oxygen transportation.

The microbial diversities during composting greatly influence conversion of lignocellulosic substrate into the biofertilizers (Jurado et al. 2014), and this species distribution plays an important role in adjusting the biochemical cycle during composting (Du et al. 2018). In this work, the succession of the functional microbial communities and metabolic functions in composting process were analyzed through high-throughput sequencing. As shown in Fig. 5b, the genera *Rhizobium* might play a key role in nitrogen fixation, whereas, the genera *Paenibacillus*, *Phenylobacterium*, *Pseudomonas* and *Pseudoxanthomonas* could make a contribution to organic matter degradation and HA biosynthesis in the rice straw composting. Therefore, the abundances of these genera significantly varied during the composting, and the changes of the bacterial community structure demonstrate that these microbes after acclimating the composting environment were gradually involved in degradation of the organic substrate and subsequent biosynthesis of HA. With the progress of composting, the relative abundance of *Rhizobium* (phylum Proteobacteria) increased, while the relative abundance of *Paenibacillus* (phylum Firmicutes) gradually decreased. Zhong et al. (2020) reported that the biological nitrogen fixation of *Rhizobium* mainly occurred at the thermophilic and mature stages during dairy manure composting. *Paenibacillus* was considered to be able to degrade lignocellulosic materials by secreting cellulase, xylanase, lignin peroxidase and laccase (Zhang et al. 2019), and possessed an excellent thermotolerance during composting (Weselowski et al. 2016). *Pseudomonas* (phylum Proteobacteria), *Phenylobacterium* (phylum Proteobacteria) and *Pseudoxanthomonas* (phylum Proteobacteria) were observed in M02 group. Nikel and de Lorenzo (2018) considered that *Pseudomonas* played a role in removing nitrogen and phosphorus in waste treatment and was more tolerant to high temperature and heavy metals than other fungi (Ghosh et al. 2019). *Phenylobacterium* can degrade part macromolecular organic matter (Weon et al. 2008), and promote HA biosynthesis. Meng et al. (2019) found that *Pseudoxanthomonas* (phylum Proteobacteria) possessed a good capacity to degrade cellulose, and made a significant contribution to phosphorus removal during cattle manure composting.

To further reveal the roles of metabolic functions of these bacteria in the process of composting, the bacterial function profiles were predicted through KEGG in this work. Glycolysis/gluconeogenesis, fructose and mannose metabolism and pyruvate metabolism showed higher relative abundances levels in M0 than those of other samples (Fig. 7). At the initial stage of composting, the easily-degradable substances in the compost would have been preferentially utilized by the microbes, following that, the macromolecular organic matter was degraded during the composting (Sanchez-Monedero et al. 1999). The differences in the abundance of these genes related to amino acid metabolism indicates that amino acid metabolism could facilitate proliferation and metabolism of the microbes by providing them with amino acids as the energy and carbon source (Lopez-Gonzalez et al. 2015). Zhang et al. (2016a) found that amino acid metabolism in bacteria affects the synthesis of humic substance. As for metabolism of cofactors and vitamins, several studies have shown that *Pseudomonas* could use vitamins as the sources of C, N and energy, and it also could improve the stability and maturity of composting (McCormick 2003; Ventorino et al. 2016). The abovementioned data further indicate that high abundances of the functional genes in these pathways may play a key role in lignocellulose biodegradation and HA biosynthesis during the rice straw composting.

In this work, the influences of different operating conditions on the biosynthesis of HA were systematically investigated using rice straw as substrate. The maximal HA yield (356.9 g kg^−1^) was obtained under the optimal inoculation size 20%, substrate particle size 0.83 mm and AR 0.3 during the composting of rice straw. In addition, the succession of the microbial community structures during the composting of rice straw were evaluated through high-throughput sequencing. The result demonstrates that Proteobacteria, Firmicutes, Bacteroidetes and Actinobacteria were the dominant phyla, affecting the humification process of composting. The metabolic function profiles of bacterial community further indicates that these functional genes in carbohydrate metabolism and amino acid metabolism were involved in lignocellulose biodegradation and HA biosynthesis. Our above findings can provide an insight into elucidating the potential functions of the dominant genera in rice straw composting and the mechanisms of HA biosynthesis.

## Data Availability

Data will be made available on request.
